# Prognostic factors of the short-term outcomes of patients with hepatitis B virus-associated acute-on-chronic liver failure

**DOI:** 10.6061/clinics/2017(11)07

**Published:** 2017-11

**Authors:** Qing Lei, Kangjian Ao, Yinhua Zhang, Deqiang Ma, Deping Ding, Changzheng Ke, Yue Chen, Jie Luo, Zhongji Meng

**Affiliations:** I Department of Infectious Diseases, Taihe Hospital, Hubei University of Medicine, Hubei, Shiyan, 442000, China; II Institute of Biomedical Research, Taihe Hospital, Hubei University of Medicine, Hubei, Shiyan, 442000, China; III Center for Evidence-Based Medicine and Clinical Research, Taihe Hospital, Hubei University of Medicine, Hubei, Shiyan, 442000, China

**Keywords:** Hepatitis B Virus, Acute-on-Chronic Liver Failure, Prognosis

## Abstract

**OBJECTIVE:**

To investigate the impact of the baseline status of patients with hepatitis B virus-associated acute-on-chronic liver failure on short-term outcomes.

**METHODS:**

A retrospective study was conducted that included a total of 138 patients with hepatitis B virus-associated acute-on-chronic liver failure admitted to the Department of Infectious Diseases, Taihe Hospital, Hubei University of Medicine, from November 2013 to October 2016. The patients were divided into a poor prognosis group (74 patients) and a good prognosis group (64 patients) based on the disease outcome. General information, clinical indicators and prognostic scores of the patients’ baseline status were analyzed, and a prediction model was established accordingly.

**RESULTS:**

Elder age, treatment with artificial liver support systems and the frequency of such treatments, high levels of white blood cells, neutrophils, neutrophil count/lymphocyte count ratio, alanine aminotransferase, gamma-glutamyl transferase, total bilirubin, urea, and prognostic scores as well as low levels of albumin and sodium were all significantly associated with the short-term outcomes of hepatitis B virus-associated acute-on-chronic liver failure. The predictive model showed that logit (*p*) = 3.068 + 1.003 × neutrophil count/lymphocyte count ratio - 0.892 × gamma-glutamyl transferase - 1.138 × albumin - 1.364 × sodium + 1.651 × artificial liver support therapy.

**CONCLUSION:**

The neutrophil count/lymphocyte count ratio and serum levels of gamma-glutamyl transferase, albumin and sodium were independent risk factors predicting short-term outcomes of hepatitis B virus-associated acute-on-chronic liver failure, and the administration of multiple treatments with artificial liver support therapy during the early stage is conducive to improved short-term outcomes.

## INTRODUCTION

Acute-on-chronic liver failure (ACLF) refers to a group of complex clinical syndromes characterized by acute severe liver function damage (caused by a number of acute triggering factors) in patients with chronic liver disease, complicated by the failure of one or more organs. ACLF is the most common type of liver failure in China and has a high mortality rate of 50-90% 1. The predominant causes of ACLF differ in different parts of the world. In China, the most important cause is infection with hepatitis virus, especially hepatitis B virus (HBV) 2. The pathogenesis of HBV-ACLF has not been well elucidated, although the “three-shock” hypothesis (i.e., immune injury, ischemia and hypoxia and endotoxin-induced damage) is currently the most generally recognized hypothesis 3.

For severe ACLF, the only effective treatment is liver transplantation. However, due to the shortage of resources, the demand for liver transplantation cannot be met. At present, the treatment strategy for HBV-ACLF is comprehensive combination with internal medicine, anti-viral medications and artificial liver support therapy 4. Artificial liver support therapy can temporarily replace part of the liver function and prevent further exacerbation of liver failure by removing toxic substances and metabolites from the serum, improving the microenvironment for liver cell regeneration and liver function repair. However, the efficacy of artificial liver support therapy for liver failure remains controversial 5-7.

The early identification, accurate diagnosis and prognostic evaluation of ACLF can provide a guiding basis for active and effective treatment. Therefore, a better understanding of prognostic factors and more precise prognostic evaluation systems for ACLF are in urgent need. A variety of factors can affect the progression and prognosis of ACLF. Many prognostic scoring systems are available for predicting the outcomes of ACLF, including the Child-Turcotte-Pugh (CTP) system, the model for end-stage liver disease (MELD) and the MELD-sodium (MELD-Na). Each scoring system has certain limitations given that not all of the influencing factors can be included in the individual assessment. The integrated MELD (iMELD) is a new scoring system that features the addition of two independent ACLF prognostic risk factors, age and serum Na levels, to the MELD scoring system 8. In addition, the albumin-bilirubin (ALBI) grading system is a recently developed scoring system to assess liver function 9. Several studies have compared the predictive ability of different scoring systems in ACLF 10-12. Comparisons of the predictive abilities of the CTP, MELD, MELD-Na, iMELD and ALBI scoring systems for the prognosis of HBV-ACLF are rarely reported. Attempts to analyze the impact of clinical parameters and the combined prognostic abilities of these parameters on ACLF prognosis have provided inconsistent results due to differences in study subjects, study phases and follow-up periods 13-15. The integration of general information, clinical indicators and a prognostic scoring system may better predict the short-term outcomes of patients with HBV-ACLF.

In this study, a retrospective investigation was carried out in HBV-ACLF patients admitted to the Department of Infectious Diseases, Taihe Hospital, Hubei University of Medicine from November 2013 to October 2016. General patient information, laboratory indicators and prognostic scores at baseline of HBV-ACLF patients with different prognoses were analyzed, and the factors that influenced the short-term outcomes of HBV-ACLF were investigated.

## PATIENTS AND METHODS

### Patient selection

Based on the clinical data in the medical record system of Taihe Hospital, Hubei University of Medicine, a retrospective analysis was conducted in patients admitted to the Department of Infectious Diseases from November 2013 to October 2016 who met the criteria of HBV-ACLF during their hospitalization. The data from the medical records of the selected patients were input in the form of case reports and verified with the clinical data system in our hospital. All of the patients were given comprehensive supportive treatment of internal medicine after admission to the hospital, including anti-viral therapy with nucleoside analogs. Artificial liver support therapy was optional based on the patient’s condition and willingness. The end point of the observation in this study was the time of discharge or in-hospital death of the patient. The study protocol was approved by the Ethics Committee of Taihe Hospital, Hubei University of Medicine.

### Inclusion and exclusion criteria

The diagnostic criteria for HBV-ACLF were based on the “Guidelines for the Prevention and Treatment of Chronic Hepatitis B” of China issued in 2015 and the “Guidelines for Diagnosis and Treatment of Liver Failure (2012 Edition)” of China. The exclusion criteria included patients complicated with other forms of viral hepatitis, alcoholic liver disease, autoimmune liver disease, drug-induced liver injury, associated tumors or severe organ disease other than hepatitis B.

### General information

The general information included gender, age, with/without (w/wo) liver cirrhosis, rebound after withdrawal of anti-viral drugs, complication with ascites at admission and the acceptance and frequency of artificial liver support therapy. All of this information was retrieved from the medical record system of Taihe Hospital.

### Clinical indicators

The following measurements were performed using venous blood collected on the first day of admission or on the morning following admission: baseline white blood cell count (WBC), absolute neutrophil count (NE), absolute lymphocyte count (LY), NE:LY ratio (NE/LY), platelet count (PLT), levels of alanine aminotransferase (ALT), aspartate aminotransferase (AST), gamma-glutamyltransferase (GGT), albumin (Alb), total bilirubin (TBil), urea (Urea), and creatinine (Cr), prothrombin time (PT), prothrombin activity (PTA), activated partial thromboplastin time (APTT), the international normalized ratio (INR) and serum Na levels. All results were retrieved from the clinical database.

### Prognostic score

The prognostic scoring systems included the CTP, MELD, MELD-Na, iMELD and ALBI. The CTP score is the cumulative result of the scores for five items (ascites, hepatic encephalopathy, TBil, Alb and PT extension time), with 1-3 points for each item and a maximum of 15 points 16, 17. The equation for the MELD score is as follows: 3.8 LN (TBil [mg/dL]) + 11.2 LN (INR) + 9.6 LN (Cr [mg/dL]) + 6.4 × cause (0 for cholestatic or alcoholic liver diseases and 1 for all others); the result is a rounded integer 18. The equation for the MELD-Na score is as follows: MELD + 1.59 × (135 - Na), wherein Na is 135 mmol/L if Na > 135 mmol/L and 120 mmol/L if Na < 120 mmol/L 19. The equation for the iMELD score is as follows: MELD + (0.3 × Age) - (0.7 × Na) + 100 8. The equation for the ALBI score is as follows: (log_10_TBil [µmol/L] × 0.66) + (Alb [g/L] × - 0.085) 9.

### Prognostic criteria

The prognostic criteria in this study were based on the ACLF clinical improvement criteria in “Guidelines for Diagnosis and Treatment of Liver Failure (2012 Edition)” of China 2. A good prognosis needs to meet all of the following conditions simultaneously: 1) the clinical symptoms are significantly improved and hepatic encephalopathy has disappeared; 2) signs of jaundice and ascites are significantly improved; and 3) liver function is significantly improved (TBil <5 ULN, PTA >40%). The patients who died, whose condition became more advanced, and those who still experienced ACLF were included in the poor prognosis group.

### Data processing

All data included in this study were retrieved from the electronic medical record system of Taihe Hospital and validated using the clinical data system. Cases with missing or incomplete data, such as AFP data, were not included in the study.

### Statistical analysis

Statistical Package for the Social Sciences (SPSS) 17.0 software was used for the statistical analysis. The measurement data were first tested for a normal distribution. Data that showed a normal distribution were represented as *x̅*±*s* and were analyzed using the t-test. The data that were not normally distributed were represented as medians (*P*_25_ and *P*_75_) and analyzed using the nonparametric rank sum test. The count data among different groups were analyzed using the χ^2^ test. The area under the receiver operating characteristic (ROC) curve was used to assess the predictive power of the five scoring systems for the prognosis of HBV-ACLF, and the cut-off value of the continuous variable was calculated. The method of likelihood-ratio-forward-selection in non-conditional binary logistic regression analysis was used to obtain the independent risk factors and to establish a predictive model. The threshold used for statistical significance was *p*<0.05.

## RESULTS

### Case enrollment

**Figure 1:**
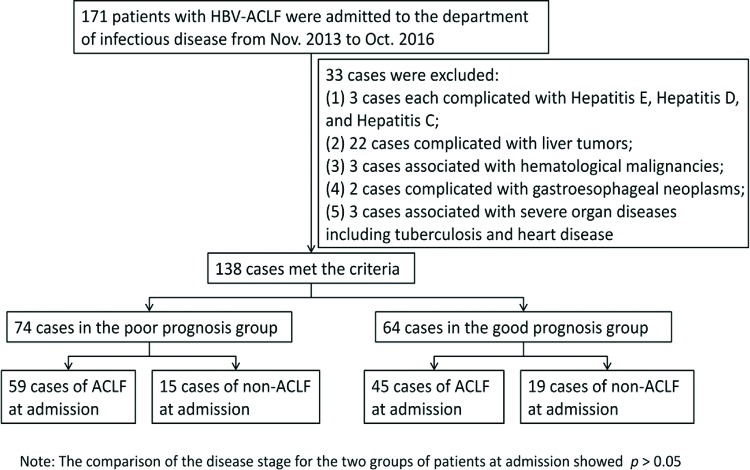
Case screening and enrollment. No statistically significant difference was observed between the poor prognosis group and the good prognosis group in terms of disease stage at admission.

A total of 171 patients with HBV-ACLF were admitted to the department of infectious disease of Taihe Hospital from November 2013 to October 2016. A total of 138 patients were selected according to the inclusion and exclusion criteria, including 111 males and 27 females with an average age of 45.80±11.01 years. There were 74 cases in the poor prognosis group, accounting for 53.6%, and 64 cases in the good prognosis group, accounting for 46.4%. Of the cases in the poor prognosis group, 59 cases showed ACLF; 15 cases were in the pre-ACLF state at admission. In the good prognosis group, the corresponding numbers were 45 cases and 19 cases, respectively (Figure 1).

### General information of the patients

The average age of the patients in the poor prognosis group was significantly higher than that in the good prognosis group (48.08±9.08 years *vs*. 43.16±12.44 years, *p*=0.01). The number of patients who received artificial liver support therapy and the frequencies of artificial liver support therapy in the good prognosis group were significantly higher than in the poor prognosis group (*p*<0.01). There were no significant differences between the two groups with respect to sex, rebound after anti-viral drug withdrawal or the complications of cirrhosis and ascites (Table 1).

### The baseline clinical indicators for HBV-ACLF patients with different prognoses

The WBC, NE, NE/LY, ALT, GGT, TBil and Urea scores/levels were significantly higher in the poor prognosis group (*p*<0.05), while the Alb and Na levels in the good prognosis group were significantly higher than in the poor prognosis group (*p*<0.01). There were no statistically significant differences between the two groups in terms of the other clinical indicators, including LY, PLT, AST, Cr, PT, APTT and INR (Table 2).

### High CTP, MELD, MELD-Na, iMELD and ALBI scores all predict poor short-term outcomes for HBV-ACLF patients

**Figure 2:**
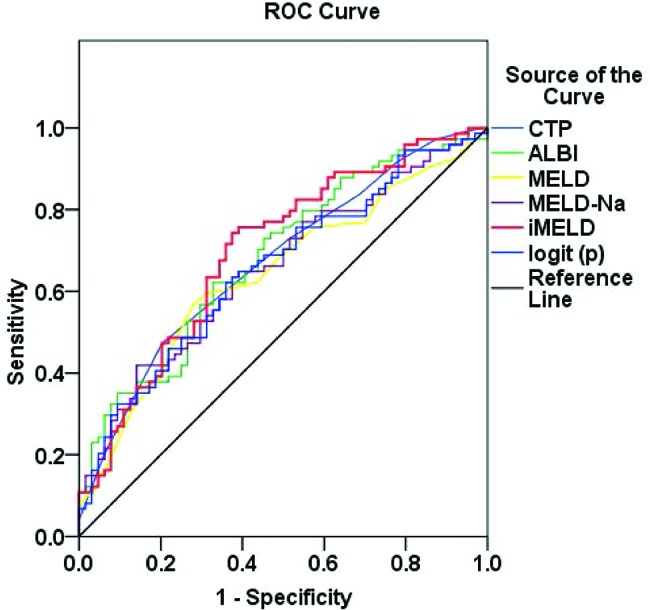
ROC curves for CTP, MELD, MELD-Na, iMELD, ALBI score and logit (*p*) curve.

Compared with the patients with a good prognosis, the patients with a poor prognosis showed significantly higher scores for the CTP, MELD, MELD-Na, iMELD and ALBI prognostic systems (*p*<0.05) (Table 3). The prognosis of HBV-ACLF was well predicted by these five types of prognostic scores. The areas under the ROC curves of the CTP, MELD, MELD-Na, iMELD and ALBI prognostic systems were 0.672, 0.641, 0.656, 0.699, and 0.682, respectively (Table 4, Figure 2).

### Independent prognostic factors and novel prediction model for HBV-ACLF

The different measures of general patient information, clinical indicators and prognostic scores at baseline for the two groups were assigned corresponding values for non-conditional binary logistic regression analysis. The cut-off values of the continuous variables were calculated using the area under the ROC curve (Tables 5 and 6) to establish the following prediction model: logit (*p*) = 3.068 + 1.003 × NE/LY - 0.892 × GGT - 1.138 × Alb -1.364 × Na + 1.651 × artificial liver support therapy. The area under the ROC curve was 0.656, with a specificity of 64.1% and sensitivity of 62.2%. NE/LY, GGT, Alb, Na and artificial liver support therapy were the independent factors influencing the short-term outcomes of HBV-ACLF (Table 7).

## DISCUSSION

HBV-ACLF is a common fatal clinical disease characterized by a large number of necrotic liver cells, a complex pathological mechanism and rapid progression. The results of this study showed that many factors are closely related to the short-term outcomes of this condition.

Age has been shown to be closely related to the severity of liver diseases and serves as an independent prognostic factor for end-stage liver disease 8, 20. Studies have found that the short-term mortality rate of patients with ACLF is positively correlated with patient age 21. In this study, the average age of the patients with a poor prognosis was significantly higher than that in the patients with a good prognosis, suggesting that age is an important prognostic factor in HBV-ACLF.

As an important supportive treatment measure for the recovery of liver function in patients with liver failure, artificial liver therapy can improve the conditions and opportunities for the regeneration of liver cells and the recovery of liver function. Artificial liver support systems take various forms, and their efficacy in treating liver failure remains controversial. Previous studies have shown that artificial liver support therapy can only improve the bilirubin level and hepatic encephalopathy of the patients, with no effect on the survival rate 5. There are also studies indicating that artificial liver support therapy can reduce the short-term mortality rate of patients with ACLF, whereas its impact on long-term survival is uncertain 6. Considering that the above results are for different types of artificial liver support therapy and different periods of application, plasma replacement therapy was primarily applied in this study. The number of patients who received artificial liver support therapy and the frequency of this therapy were significantly higher in the good prognosis group than in the poor prognosis group. Moreover, artificial liver support therapy was an independent factor for the short-term outcomes of HBV-ACLF. Therefore, the early active implementation of treatment combined with artificial liver support therapy can significantly improve the short-term outcomes of patients with HBV-ACLF.

WBC, NE and the NE/LY ratio are often associated with inflammation and infection. According to the “three-shock” hypothesis, inflammation is an important step in the development of HBV-ACLF 3. At the same time, studies have shown that infection can induce and promote the development of ACLF 22, and the severity of inflammation is significantly correlated with the prognosis of ACLF 23 The study of Li et al 13 showed that WBC is an independent risk factor for HBV-ACLF. In this study, the WBC, NE count and NE/LY ratio of the patients with a poor prognosis were significantly higher than those of the patients with a good prognosis, suggesting that, in addition to WBC, a high NE count and NE/LY ratio are also important prognostic factors for HBV-ACLF.

The following events occur in cases of liver failure: liver cell necrosis occurs; liver function is impaired; enzyme synthesis is reduced; disorders in detoxification, excretion and transport capacity occur; bilirubin, amino acids and other substances are released into the blood and accumulate; serum bilirubin and transaminase levels increase; and the synthesis of proteins and coagulation factors decreases, resulting in decreased Alb levels and the development of coagulation disorders. In the present study, liver function and coagulation function were abnormal in both groups, and the levels of ALT, GGT, TBil and Alb all showed significant differences between the two groups. Of these measures, GGT and Alb were independent risk factors for poor prognosis of HBV-ACLF. However, a comparison of the baseline PTA of the patients in the two groups showed a significance level of *p*=0.05. Therefore, this difference was not considered statistically significant. Baseline PTA may be related to the patient’s stage of disease at admission. Because the majority of patients in this study had reached the ACLF state at admission, with PTA ≤40%, the difference between the two groups was small. Changes and differences in coagulation can be further investigated and analyzed at different times and over different observation periods.

Ascites, hyponatremia and hepatorenal syndrome are common complications of ACLF. Complications such as refractory ascites, hyponatremia and renal injury are often interrelated and may continuously degenerate 2. In this study, the serum Na levels of patients in the poor prognosis group were significantly lower than those of the patients with a good prognosis. Low Na levels were an independent risk factor for poor prognosis of HBV-ACLF; this finding is consistent with the results of the studies of Zhang et al 15 and Shi et al 24. The results suggest that electrolytes should be closely monitored and electrolyte imbalances should be corrected during HBV-ACLF treatment.

In this study, the serum levels of urea were higher in the poor prognosis group than those in patients with good prognoses, whereas no significant difference in the level of Cr was found between the two groups. Accordingly, renal function damage may have a certain relationship with the prognosis of HBV-ACLF. However, the etiology of all patients included in this study was HBV infection. The diagnostic criteria for ACLF were based on the “Guidelines for Diagnosis and Treatment of Liver Failure (2012 Edition)” of China. In cases of ACLF associated with HBV infection, early organ damage is primarily manifested as liver function damage and coagulation dysfunction rather than as increased Cr levels.

Use of the prognostic evaluation system can help clinicians predict disease prognosis in the early stage of the disease and assess the severity of the disease, providing guidance in the choice of active and effective treatment. However, because each prognostic scoring system has limitations, this study for the first time compared the five scoring systems (CTP, MELD, MELD-Na, iMELD and ALBI) in HBV-ACLF patients with differing prognosis. The results showed that all five prognostic scoring systems demonstrated good predictive value for ACLF. The prognostic scores of the patients at baseline status in the poor prognostic group were significantly higher than those of the patients in the good prognosis group, suggesting that the prognosis of HBV-ACLF was significantly correlated with the prognostic scores at baseline status.

A novel prognostic scoring system was established according to the factors that affect the short-term outcomes of HBV-ACLF, as follows: logit (*p*) = 3.068 + 1.003 × NE/LY - 0.892 × GGT - 1.138 × Alb - 1.364 × Na + 1.651 × artificial liver support therapy. The sensitivity and specificity were 62.2% and 64.1%, respectively. The sensitivity of this novel predictive model was much higher than those of MELD-Na and MELD scores. Additionally, the area under the ROC curve of this predictive model was much larger than that of the MELD score, although it was smaller than that of iMELD score. Most importantly, except for serum Na, the parameters of NE/LY, GGT, Alb, and artificial liver support therapy were shown to be important factors that affect the short-term outcomes of HBV-ACLF as well. In addition to the indexes of liver function, complications such as infection were also included in this model. Thus, the novel predictive model was extremely suitable for ACLF patients in the end stage with multiple complications, especially those with an extremelyelevated INR, and this circumstance was restricted in evaluations using the iMELD, MELD-Na, and MELD scores.

Differences in study subjects, study phases and sample sizes may lead to different results. Although this study was a comprehensive study of factors that contribute to the short-term outcomes of HBV-ACLF, it had some limitations. First, all patients were enrolled from a single center, which may not reflect regional influences on the prognosis. Secondly, alpha fetal protein and a history of antiviral therapy were not examined in this study. Lastly, only short-term outcomes were analyzed. Multi-center studies over different observation periods are required to further research HBV-ACLF and to provide a better theoretical basis for its clinical diagnosis and treatment.

In conclusion, a variety of factors can affect the prognosis of HBV-ACLF patients. Older age, high WBC, NE, NE/LY, and TBil levels, renal dysfunction, hyponatremia, hypoproteinemia and high prognostic scores at baseline often suggest a poor prognosis. For patients with HBV-ACLF, within the context of anti-viral treatment and comprehensive internal medicine treatment, infection control should be strengthened and the stability of the patient’s lab values, including the electrolyte balance, should be maintained. In addition, early active treatment combined with artificial liver support therapy is recommended. Further studies are needed to enhance our understanding of HBV-ACLF pathogenesis and reduce its morbidity and mortality.

## AUTHOR CONTRIBUTIONS

Lei Q, Ke C, Chen Y, Luo J and Meng Z conceived and designed the study. Lei Q, Ao K, Zhang Y, Ma D, Ding D were responsible for the data collection. Lei Q analyzed the data and wrote the manuscript. Meng Z was responsible for the manuscript final revision.

## Figures and Tables

**Table 1:** General information of the patients.

	Good prognosis	Poor prognosis	Statistics value	*p* value
Age (years)	43.16±12.44	48.08±9.08	t′ = 2.621	0.010
Gender (male/female)	52/12	59/15	χ^2^ = 0.05	0.822
W/wo liver cirrhosis	19/45	32/42	χ^2^ = 2.707	0.100
W/wo drug-withdrawal rebound	6/5	10/22	χ^2^ = 1.035	0.309
W/wo ascites	41/23	55/19	χ^2^ = 1.707	0.191
W/wo ALST	54/10	36/38	χ^2^ = 19.31	0.000
Number of treatments	1.62 (0.79, 2.55)	0.64 (0, 1.61)	Z = -4.422	0.000

Note: W/wo indicates with/without; ALST is the abbreviation of artificial liver support therapy.

**Table 2:** Clinical indicators of the baseline status of the patients.

	Good prognosis	Poor prognosis	Statistics value	*p* value
WBC (× 10^12^/L)	5.31 (4.12, 6.79)	6.40 (4.48, 9.07)	Z = -2.291	0.022
NE (× 10^12^/L)	3.16 (2.28, 4.99)	4.03 (2.86, 6.57)	Z = -2.248	0.025
LY (× 10^12^/L)	1.13 (0.84, 1.43)	1.21±0.63	Z = -0.566	0.572
NE/LY	2.70 (1.79, 5.37)	3.96 (2.93, 6.69)	Z = -2.517	0.012
PLT (× 10^12^/L)	88.0 (65.25, 125.67)	85.0 (46.0, 122.33)	Z = -1.087	0.277
ALT (U/L)	507.5 (175.5, 902.0)	198.0 (81.0, 674.0)	Z = -2.589	0.01
AST (U/L)	289.5 (124.0, 767.5)	210.0 (95.67, 424.0)	Z = -1.543	0.123
GGT (U/L)	108.5 (62.5, 159.5)	66.0 (47.0, 125.0)	Z = -2.735	0.006
Alb (g/L)	33.74±5.174	30.71±5.56	t = -3.30	0.001
TBil (μmol/L)	234.24±106.27	301.62±144.45	t′ = 3.147	0.002
Urea (mmol/L)	4.17 (2.98, 5.44)	4.54 (3.54, 7.52)	Z = -2.002	0.045
Cr (μmol/L)	55.75 (42.7, 77.95)	56.95 (42.7, 85.5)	Z = -0.431	0.666
Na (mmol/L)	138.91±4.209	136.07±6.33	t′ = -3.136	0.002
PT (s)	22.45 (18.5, 26.03)	25.0 (18.6, 30.9)	Z = -1.738	0.082
PTA (%)	28.27 (22.64, 36.97)	26.37±12.80	Z = -1.964	0.05
APTT (s)	52.83±14.70	56.33±18.69	t′ = 1.231	0.22
INR	1.98±0.62	2.05 (1.6, 2.72)	Z = -1.642	0.101

Note: Normally distributed data are represented as *x̅*±*s*. Non-normally distributed data are represented as medians (*P*_25_ and *P*_75_).

**Table 3:** Prognostic scores of the patients at baseline.

	Good prognosis	Poor prognosis	Statistics value	*p* value
CTP score	10.11±1.78	11.2±1.68	t = 3.706	0.000
MELD score	18.08±7.10	21.58±7.39	t = 2.828	0.005
MELD-Na score	18.95±7.7	24.18±10.11	t = 3.573	0.000
iMELD score	33.79±9.15	40.75±9.90	t = 4.268	0.000
ALBI score	-1.34±0.52	-1.01±0.54	t = 3.639	0.000

**Table 4:** Comparison of the five types of prognostic scores.

	Area	Sensitivity (%)	Specificity (%)	Std. error	Asymptotic Sig.	95% C.I.	
						Lower	Upper
CTP	0.672	47.3	79.7	0.045	0.000	0.593	0.761
MELD	0.641	56.8	71.9	0.047	0.004	0.549	0.733
MELD-Na	0.656	41.9	85.9	0.046	0.002	0.565	0.746
iMELD	0.699	74.3	62.5	0.045	0.000	0.611	0.786
ALBI	0.682	62.2	67.2	0.045	0.000	0.594	0.771
Logit(p)	0.656	62.2	64.1	0.046	0.002	0.565	0.746

**Table 5:** Area under the ROC curve and the cut-off value of each variable.

	Cut-off value	c-statistic	95% CI	
			Lower	Upper
Age	43.5	0.643	0.549	0.738
WBC	6.75	0.613	0.52	0.707
NE	5.635	0.611	0.517	0.705
NE/LY	2.947	0.624	0.53	0.719
ALT	127.5	0.372	0.279	0.465
GGT	76	0.365	0.272	0.457
Alb	34.85	0.343	0.252	0.435
TBil	251.2	0.632	0.54	0.725
Urea	6.365	0.599	0.505	0.693
Na	136.3	0.361	0.268	0.454
CTP	11.5	0.672	0.593	0.761
MELD	21.448	0.641	0.549	0.733
MELD-Na	25.636	0.656	0.565	0.746
iMELD	34.705	0.699	0.611	0.786
ALBI	-1.119	0.682	0.594	0.771

**Table 6:** Variable assignment.

	Assignment	
	0	1
Age	≤ 43.5	> 43.5
WBC	≤ 6.75	> 6.75
NE	≤ 5.635	> 5.635
NE/LY	≤ 2.947	> 2.947
ALT	≤ 127.5	> 127.5
GGT	≤ 76	> 76
Alb	≤ 34.85	> 34.85
TBil	≤ 251.2	> 251.2
Urea	≤ 6.365	> 6.365
Na	≤ 136.3	> 136.3
MELD	≤ 21.448	> 21.448
MELD-Na	≤ 25.636	> 25.636
iMELD	≤ 34.705	> 34.705
CTP	≤ 11.5	> 11.5
ALBI	≤ -1.119	> -1.119
Artificial liver support therapy (ALST)	No	Yes
Prognosis	Good prognosis	Poor prognosis

**Table 7:** Logistic regression analysis results.

	B	SE	Wald	Sig	Exp(B)	95% CI for Exp(B)	
						Lower	Upper
NE/LY	1.003	0.444	5.109	0.024	2.728	1.143	6.512
GGT	-0.892	0.428	4.338	0.037	0.41	0.177	0.949
Alb	-1.138	0.511	4.951	0.026	0.321	0.118	0.873
Na	-1.364	0.455	8.979	0.003	0.256	0.105	0.624
Artificial liver support therapy	1.651	0.473	12.161	0.000	5.21	2.06	13.174
Constant term	3.068	1.441	4.535	0.033	21.489		
